# Retrospective Cohort Study Evaluating Time to Graft Between Intact Fish Skin Grafts and a Synthetic Matrix Used in Complex Full-Thickness Acute Traumatic and Burn Wounds

**DOI:** 10.3390/ebj7030038

**Published:** 2026-07-13

**Authors:** David M. Hill, Muntazim Mukit, Kais Atmeh, Karapet Davtyan, Dani Kruchevsky, Xiangxia Liu, Mahmoud Hassouba

**Affiliations:** 1 Department of Pharmacy, Regional One Health, Memphis, TN 38103, USA; 2 Department of Plastic Surgery, University of Tennessee Health Science Center, Memphis, TN 38163, USA; mmukit@uthsc.edu (M.M.); katmeh86@gmail.com (K.A.); kdavtyan@uthsc.edu (K.D.); dkruchev@uthsc.edu (D.K.); liuxiangxia@gmail.com (X.L.); mhassoub@uthsc.edu (M.H.)

**Keywords:** burns, skin transplantation, skin, artificial, wound healing, heterografts, intact fish skin graft, xenografts

## Abstract

**Highlights:**

**What are the main findings?**

- Intact fish skin grafts and a synthetic matrix can be deployed successfully in complex full-thickness acute traumatic and burn wounds.
- Use of intact fish skin grafts was associated with a quick time to a graftable wound bed.

**What are the implications of the main findings?**

- Data support expanding the armamentarium deployed to successfully close full-thickness acute traumatic and burn wounds.
- Data support conducting a prospective, randomized controlled study.

**Abstract:**

**Introduction**: While global burden is underappreciated, traumatic wounds account for 23 million annual emergency department visits in the United States, accounting for trillions in medical costs and work and quality of life loss. We hypothesized that the use of intact fish skin grafts (IFSGs) would result in faster time to obtain a graftable wound bed compared to a synthetic matrix (SM). **Methods**: This retrospective analysis of patients with full-thickness acute traumatic wound was conducted on a matched sample of patients receiving IFSG versus SM. We hypothesized that there would be at least a 10-day difference between the two treatments. Two surgeons reviewed electronic charts for primary outcomes. Incomplete charts were excluded. For sensitivity analysis, both simple linear regression and generalized linear mixed models were utilized to adjust the primary outcome to potential confounders. **Results**: Data were reviewed for 80 patients. The final cohort included 38 patients [IFSG (*n* = 12) and SM (*n* = 26)]. Demographics were not different between groups. The mean age was 42.4 ± 20.7 years, with 62.2% being male and equal proportions having burns treated vs. another traumatic wound. The most common site was the lower extremity. All IFSG patients had negative pressure wound therapy (NPWT) placed at the time of implantation vs. 50% of SM (*p* = 0.001). The SM group had a larger TBSA (*p* < 0.001) and wound size (*p* = 0.001). Differences were subsequently used to adjust primary outcomes via regression. Patients with IFSG had faster time to a graftable wound bed compared to SM (mean ± SD: 13.9 ± 5.3 vs. 29.1 ± 10.3, *p* < 0.001). Neither wound size, burn severity, presence of infection, nor use of NPWT significantly altered the inference. **Conclusions**: In this paired sample, the use of IFSG as a dermal template for full-thickness traumatic injuries was associated with faster time to obtain a graftable wound bed compared to an SM. However, regression analysis alone is not enough to conclude that the same difference would exist in a prospectively controlled experiment, which should be performed.

## 1. Introduction

It is estimated that nearly 11 million burn injuries occur worldwide each year [1]. The true worldwide incidence of non-fatal acute traumatic injuries is difficult to estimate [2]. It is estimated that in the United States alone, traumatic wounds account for 23 million emergency department visits, 3.5 million hospital admissions, and an estimated $1.92 trillion lost in medical costs and work and quality-of-life loss [3,4]. In 2020, the burden of injury prevalence was estimated to account for over $4.6 trillion, or 22% of the US gross domestic product [3]. In 2020, there were 698,555 burn-related insurance claims [5]. As providers, our goal is to return patients to their pre-injury status as quickly as possible. When acute traumatic wounds are involved, our goal includes closure of the wound, as quickly as is feasible, while managing the many complexities involved in the patients’ care.

There are many options to aid the closure of complex traumatic wounds, all with unique properties contributing to their intended deployment [6]. An important aspect to consider is the time required for complete wound closure. We hypothesized that the use of intact fish skin grafts (IFSGs) as a dermal substitute would result in faster time to obtain a graftable wound bed compared to a synthetic matrix (SM). We aimed to compare the subjective number of days between dermal substitute placement and the date the wound bed was deemed ready to receive an autograft.

## 2. Methods

### 2.1. Design

The study was a single-center, retrospective cohort study. Patients admitted to the American Burn Association-verified burn center between July 2019 and April 2024, and who received the IFSG or SM, were eligible to be screened for inclusion. Initial inclusion required age of at least 18 years, the surgery and wound to be managed by the Burn or Plastic Surgery teams, and presence of a full-thickness wound. Patients were excluded if they were pregnant or incarcerated or the chart was incomplete, such as unable to determine the primary endpoint (i.e., date wound bed determined ready to receive skin graft).

### 2.2. Experimental Protocol

All of the cases were completed and discharged prior to initiation of this retrospective study. Dual Institutional Review Board approval was secured prior to beginning this work. The IFSG (Kerecis GraftGuide^®^, Kerecis, LLC, Ísafjörður, Iceland) was a xenograft created from minimal processing of Atlantic cod, approximating 1.7 mm thick. The SM (Novosorb^®^ BTM, PolyNovo Biomaterials Pty Ltd, Port Melbourne, Victoria, Australia) utilized as a comparator was a 2 mm thick, biodegrading polyurethane foam bonded to a perforated sealing membrane.

Patients and wounds, where IFSG was used and met the inclusion/exclusion criteria, were matched 1:3 to a comparable control (i.e., SM) in a reverse-chronological selection (e.g., most recent injury screened first). All operations were performed by one of three attending surgeons. Patients and wounds were matched according to patient age (±5 years) and type of acute wound (traumatic or burn). As SM had a longer history of use, it was anticipated that there would be more SM cases, which informed the feasibility and design. Attempting to avoid eliminating too many cases from the analysis, we limited variables during the matching process, necessitating regression analysis.

The primary outcome of time to a graftable wound bed was the number of days until the wound bed was deemed ready to receive a skin graft. Readiness to receive a skin graft was determined using the following criteria: (1) beefy red granulation or pale pink-to-red blanching tissue present in approximately 95% of the target wound bed; (2) lacking signs or symptoms of ongoing infection; (3) minimal fibrinous drainage. Subjectivity was reduced by utilizing dual independent review. The number of days was determined independently by two surgeons through retrospective chart review, which included progressive, periodic pictures and daily wound care notes housed within the electronic health record. The surgeons were blind to each other’s assessments but not treatment allocation due to the retrospective nature of the study. In the event of disagreement, a third surgeon served as arbiter. There was only disagreement with one patient, producing an excellent intraclass correlation coefficient of 0.999 (95% CI 0.997–0.999).

### 2.3. Data Collection

Along with the primary outcome, we collected demographics, injury etiology, and characteristics for each patient. We collected wound details necessary to determine severity, depth, presence of exposed structures, size, location, and post-operative use of negative pressure wound therapy (NPWT). We collected length of stay, but this number should be interpreted with caution, understanding that there are far more reasons patients may have a delayed discharge than the individual treated wound within this sample. This fact also informed the selection of the studied primary outcome. It was postulated that some patients would be discharged in the days between dermal template placement and subsequent skin graft placement, which informed the exclusion criteria and initial sample size. Days to wound excision, substitute placement, and autograft were collected. Related, we collected the operative procedures preceding IFSG or SM placement. Clinical details included infection, the use of topical and systemic antibiotics, and the presence of shock or vasopressor use.

### 2.4. Sample Size Determination

Based on the existing literature and anecdotal experience, we estimated there to be at least a 10-day difference between the two treatments, regarding the primary outcome [7,8]. In order to properly test this hypothesis, we planned to screen and enroll a matched sample. Using 80% power (alpha = 0.05), a 1:1 matched design would require 32 patients, while a 1:2 and 1:3 match would require 36 and 43 patients, respectively. A 14-day difference would only require 18 total patients (9 in each group). After matching, we aimed to include 80 patients to ensure each patient with IFSG had at least 1 match after exclusion criteria were applied. This would allow our final sample to be appropriately powered after exclusion criteria were applied. We anticipated that the sample would have some missing data after final chart review, which would preclude matching into the final sample and informed the a priori sample size determination.

### 2.5. Statistical Analysis

Demographic data was analyzed by Fisher’s exact test, if dichotomous. Continuous data was compared using either Student’s *t*-test or the Mann–Whitney U test, depending on normality determined via visual inspection of the data and the Kolmogorov–Smirnov test. The primary outcome of time to graftable wound bed was initially analyzed using Student’s *t*-test. Subsequently, the primary outcome was analyzed by the log-rank test and graphically depicted via a Kaplan–Meier plot. Values for the primary outcome were censored on day 60 and the day of skin substitute failure. Finally, the primary outcome was analyzed via simple linear regression and generalized linear mixed modeling, including covariates found to be both different during univariate analysis and clinically important. For example, infection occurring after dermal substitute placement was included in the model to test its independent association with healing time and perform a sensitivity analysis. As matching was conducted based on age and acute wound type, we anticipated the groups to be clinically similar and the two variables were not included in the modeling. However, there were a few variables that could have biased the model and required further exploration. Variables resulting in a *p* value < 0.1 during univariable analysis were considered for mixed modeling. Variable inflation factor, Mallows C(p) statistic, R-square, and *p* value were all considered during simple linear regression. Statistical analysis was performed in SAS 9.4 under a current university license.

## 3. Results

**Figure 1:**
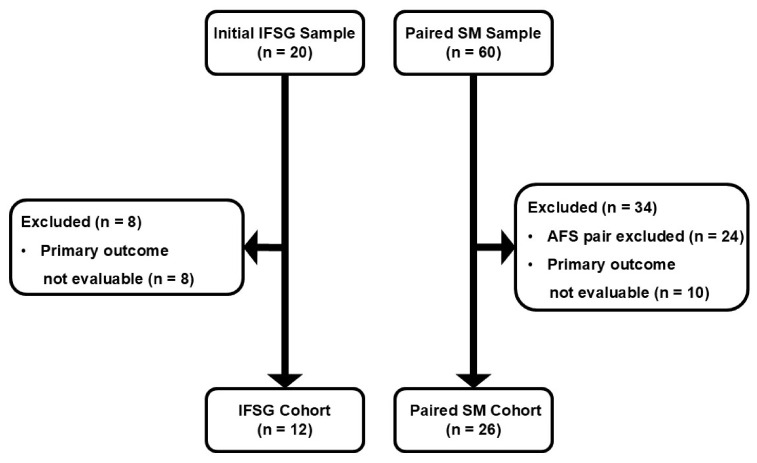
Each of twenty patient cases where intact fish skin graft (IFSG) was utilized to repair a full-thickness traumatic or burn wound was paired (1:3), with a corresponding case where a synthetic matrix (SM) was utilized instead. The cases were paired based on wound type and patient age. Cases (and pairs) were subsequently dropped from analysis if the primary outcome could not be determined, based on retrospective chart review, or if the corresponding IFSG pair was excluded.

Twenty patients were initially included, where IFSG was selected to repair a full-thickness deficit, and paired with 60 patients that had SM chosen for their management (Figure 1). As part of the data collection, each of the 80 cases, including electronic health records documentation and clinical media (i.e., wound photographs), was reviewed for determination of the primary outcome. Two surgeons independently reviewed each case. Eight of the IFSG cases were excluded due to inability to determine the definitive date of wound bed readiness. As such, their SM pairs were excluded (*n* = 24). The date of event (i.e., wound bed readiness) could not be determined for 10 additional SM cases, which were excluded. The final cohort included 12 IFSG cases and 26 SM cases (Figure 1).

Considering all 38 patients, 62.8% were male and 50% were Caucasian. The cases were evenly distributed between acute burn and other acute traumatic wounds. Demographics were similar between cohorts (Table 1), except substance abuse was more common in the IFSG group. Due to the pairing strategy, age and wound type were similar between groups, as were sex and race. Wound site and presence of exposed structure (e.g., bone, tendon, muscle) were also not different (Table 1). Percentage of total body surface area (2.1 ± 1.8% versus 28.8 ± 14.4%, *p* < 0.001) and wound size [130.0 (60.0, 394.0) cm^2^ versus 565.5 (332.0, 1771.0) cm^2^, *p* = 0.001] significantly differed between cohorts. Additionally, every IFSG case had negative pressure wound therapy (NPWT) applied over the matrix, whereas only half of the SM group had adjunctive NPWT (*p* = 0.003).

**Figure 2:**
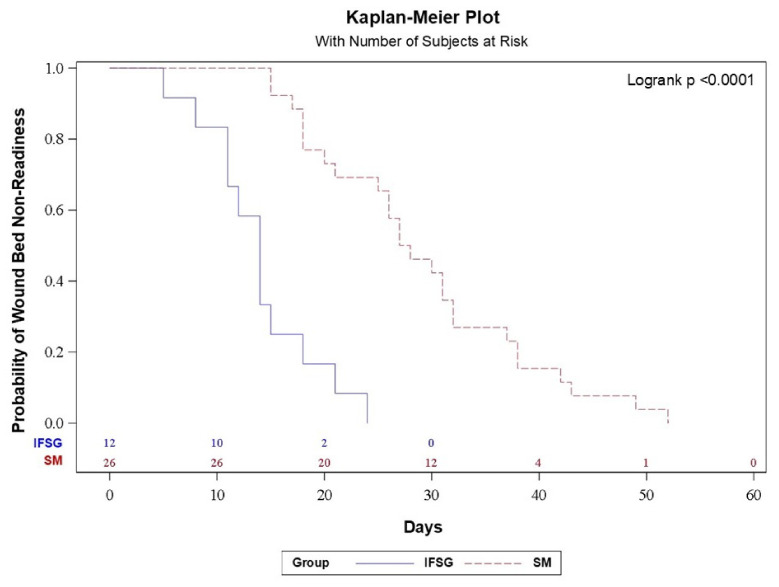
Kaplan–Meier plot comparing time from dermal substitute application to wound bed readiness to receive autograft (i.e., time to obtain a graftable wound bed) between intact fish skin grafts (IFSGs) and synthetic matrix (SM). Patients were censored at day 60, though no patient required censoring. The top left of the graph depicts that all patients treated with IFSG (*n* = 12) and SM (*n* = 26) in this cohort had a 100% probability of not being ready to receive an autograft. The groups quickly diverged (*p* < 0.0001). The bottom row depicts the number of patients in each group remaining at each time point that had not yet experienced the event (i.e., a wound that was ready to autograft).

Regarding the primary outcome, IFSG was associated with a significantly faster time (Figure 2) to a graftable wound bed [−15.2 days (95% CI −20.3, −10.0), *p* < 0.001] compared to the paired SM-treated wounds [13.9 ± 5.3 days versus 29.1 ± 10.3 days, *p* < 0.001]. In the matched sample, there were more infections noted in the SM cohort, though too few for statistical significance [0 versus 5 (19.2%), *p* = 0.158]. Regarding infection, two were managed with systemic antimicrobials, one topically, and two with both routes. Four had culture results, all being polymicrobial. *Staphylococcus* sp. was present in three, *Pseudomonas* sp. in three, *Enterobacteriacae* sp. in one, and *Acinetobacter* sp. in one. All 38 cases were considered successes, with only one patient in the SM group noting partial failure (e.g., 90% take). Time to initial wound excision was skewed but was not statistically different between IFSG and SM [4.0 (0.5, 18.0) days versus 1.0 (0, 3.0) days, *p* = 0.237]. Not considering potential confounders, time to split-thickness skin graft (STSG) was faster in the IFSG cohort [14.0 (11.0, 16.5) days versus 36.0 (30.0, 39.0) days, *p* < 0.001]. Length of stay can also be confounded but was not different under univariate analysis [19.0 (7.5, 45.5) days versus 26.0 (17.0, 49.0) days, *p* = 0.177].

## 4. Discussion

**Figure 3:**
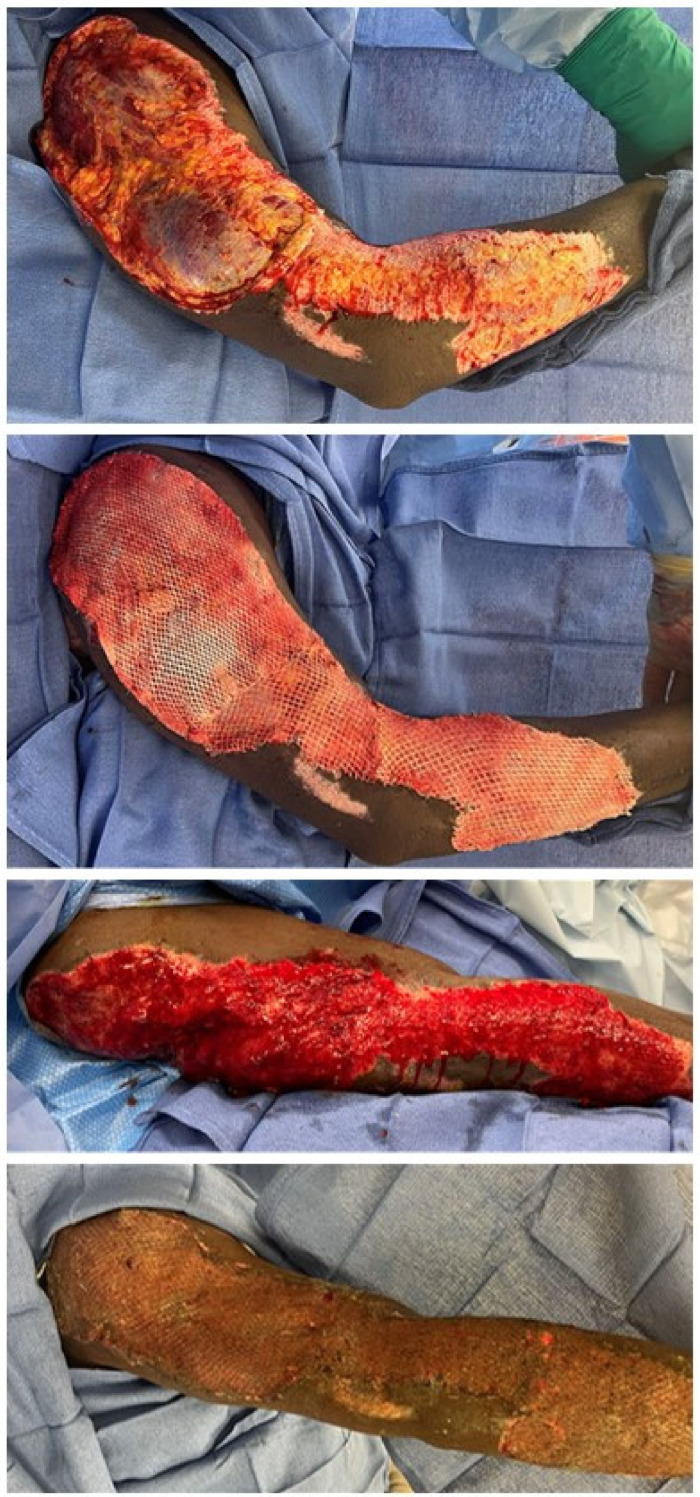
Progression of a full-thickness burn wound treated with intact fish skin grafts (IFSG) and subsequent autograft. (**Top**) Day 0, immediately post burn wound excision. (**Top–middle**) Day 0, post IFSG application. (**Bottom–middle**) Day 7, post IFSG application: deemed ready for autograft transplant. (**Bottom**) Five days following autograft application.

**Figure 4:**
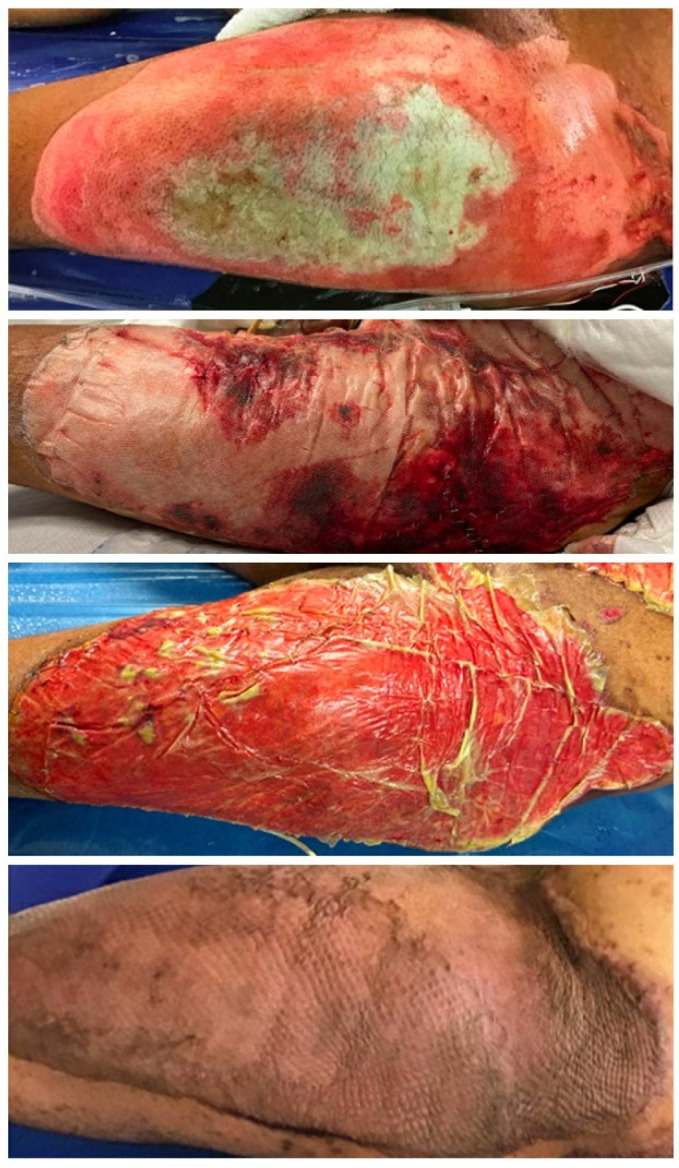
Progression of a full-thickness burn wound treated with synthetic matrix (SM) and subsequent autograft. (**Top**) Day 0, prior to burn wound excision. (**Top–middle**) Day 1, post SM application. (**Bottom–middle**) Day 25, post SM application: deemed ready for autograft transplant. (**Bottom**) Four months following autograft application.

Acute traumatic full-thickness wounds can be clinically challenging to close, especially those with complex comorbidities and social barriers. There are many biologic and synthetic dermal matrices that can be deployed to assist with wound closure, though few product comparisons are published. This retrospective study was the first to compare use and outcomes of fish skin and a synthetic matrix in a mixed trauma population. The review included a demographically diverse population. Both the IFSG (Figure 3) and SM (Figure 4) were successfully deployed in this sample of complex wounds.

The size of the overall injury and treatment site is clinically important and could have contributed to the difference noted in the primary outcome. Selection bias cannot be ruled out. Given the resource-limited, retrospective design, it is not possible to determine the exact reason that there were differences in NPWT used. Due to differences in wound size and treatment noted in the univariate analysis, both simple linear regression and generalized linear mixed modeling were utilized to consider possible confounding, and for sensitivity analysis, time from dermal substitute implantation to graftable wound bed was used as the response variable. During linear regression analysis, use of NPWT, wound size, TBSA, and incidence of infection post-application were considered in addition to treatment group (Table 2). Given 38 observations, a multivariable model could reliably include up to four variables. Target wound size was a stronger predictor than TBSA. The final, iteratively reduced model included treatment group, wound size, and infection. Keeping wound size and presence of infection constant, the use of IFSG was associated with more than a 15-day reduction in time to a graftable wound bed, when compared to SM. The inference was nearly identical after mixed modeling [−16.1 (95% confidence interval: −21.8, −10.4) days, *p* <0.0001]. In the mixed model, treatment group, wound size, and infection were included as fixed variables. Variability between pairs and correlation within pairs were considered using random effects. Adjusting the model by considering TBSA and use of NPWT had no significant effect. Future prospective work can eliminate the need for regression analysis by controlling for differences in wound size and use of NPWT during the design and enrollment phases. Although a randomized controlled trial will require significant resources, the findings of this study support the need to explore further.

All the cases included from both groups were single applications with subsequent skin grafting. All were considered successes, which is noteworthy given the diverse population included. Nearly half had a significant past medical history. Over half had a significant substance use history. Seven patients had a history of diabetes with an average Hemoglobin A1c of 7.6 ± 1.1. There were numerically more patients with diabetes in the IFSG group than the SM group (Table 1), which should have favored the SM group. Glycemic control post-application was not collected, though the meticulous care dedicated to glycemic control within our center is well published as ongoing performance improvement initiatives. Nearly half had the matrices applied over an exposed structure. Few patients in the overall sample had infections post-application, which reduced necessary systemic antimicrobial exposure. Application over bone was more common in the IFSG group, while muscle/tendon was more common in the SM group, though neither difference met statistical significance.

This report should be interpreted within the context of its limitations. Due to the study being retrospective, it is not possible to determine if the chosen dermal substitute was the reason for faster time to a graftable wound bed. As stated above, selection bias could have been present in this retrospective analysis, and a regression analysis alone cannot completely eliminate this possibility. Measurement bias was possible, as it was not possible to blind the surgeon to treatment allocation during chart review. The choice in primary outcome (time to graftable wound bed instead of time to autograft) in this study was based on balancing usefulness and feasibility. While time to autograft placement is clinically important, barriers to final autografting exist and can be confounded by many variables, especially with large cases. Exact reasons are difficult to capture in retrospect from the electronic health record. For example, there may be patients with limited donor sites that will inhibit subsequent autograft placement. Similarly, operating theaters may not be available at the exact time the wound bed is ready. Or sometimes, patients have substitutes placed to temporize the wound and are discharged to another level of care, while the wound bed matures or donor sites can prepare for reharvesting. In such cases, there may be social barriers to readmission and subsequent grafting, or several other reasons grafting was delayed unrelated to the readiness of the treated wound. Also, there was an imbalance in the groups regarding the use of NPWT. NPWT has been shown to accelerate granulation when used over dermal substitutes applied to burn wounds [9,10,11]. Additionally, there were differences in the size of wound burden between the cohorts, which may be clinically meaningful. We considered this in advance and augmented our univariate comparison with two different methods of regression analysis. The difference in primary outcome was consistent through each. Due to missing data for some of the patients screened, we had to exclude many, but this possibility was planned for during the initial sample size considerations. Though we powered the study to find a 10-day difference, we found a larger difference than was planned. Only 14 patients would have been needed to give an adequate chance to find a 15.2-day difference in the primary outcome, which was our adjusted finding. A post hoc power analysis determined that the study had a power of 99.8%. The post hoc power analysis was performed for reference only, and should be interpreted with caution. A larger-scale prospective trial is needed to truly determine whether the difference between groups is due to treatment allocation.

## 5. Conclusions

There are many factors to consider when choosing a dermal substitute to assist with full-thickness wound closure. Patient demographics and social barriers must be considered in conjunction with the traumatic injury. In this analysis, we demonstrate the use of both IFSG and an SM for acute full-thickness burns and other traumatic injuries in a complex cohort. The differences seen between IFSG and SM were from retrospective data and required significant adjustment due to differences in injury size and use of NPWT. It may not be reasonable to infer the same findings with larger wounds from this data alone. We present data supporting the need for a larger, well-controlled randomized study and results demonstrating that IFSG can be successfully deployed in traumatic full-thickness injuries, including burns.

## Figures and Tables

**Table 1:** Demographics and injury characteristics.

Variable	IFSG (*n* = 12)	SM (*n* = 26)	*p*
Age, years ^a^	38.5 (22.0, 63.5)	34.5 (24.0, 65.0)	1.000
Male ^b,c^	7 (63.6)	16 (61.5)	1.000
Race ^b^			0.692
Caucasian	6 (50.0)	13 (50.0)	
Black	4 (33.3)	11 (42.3)	
Other	2 (16.7)	2 (7.7)	
Wound Type ^b^			1.000
Burn	6 (50.0)	13 (50.0)	
Trauma	6 (50.0)	13 (50.0)	
Albumin, g/dL ^d^	3.8 ± 0.4	3.4 ± 0.4	0.102
Prealbumin, mg/dL ^d^	19.1 ± 8.3	17.2 ± 6.9	0.557
C-reactive protein, mg/dL ^a^	4.1 (2.5, 6.1)	2.0 (1.4, 2.7)	0.376
Past Medical History ^b,e^	7 (58.3)	11 (42.3)	0.569
Hypertension	6 (50.0)	5 (19.2)	0.119
Diabetes	3 (25.0)	4 (15.4)	0.656
CAD/HLD	1 (8.3)	4 (15.4)	1.000
PVD/clotting disorder	0	3 (11.5)	0.538
Chronic kidney disease	1 (8.3)	0	1.000
Social History ^b,e^	9 (75.0)	12 (46.2)	0.161
Tobacco	5 (41.7)	12 (46.2)	0.796
Alcohol	6 (50.0)	2 (7.7)	0.007
Marijuana	2 (16.7)	2 (7.7)	0.577
Methamphetamines	0	2 (7.7)	1.000
TBSA burned, % ^d^	2.1 ± 1.8	28.8 ± 14.4	<0.001
Wound Size, cm^2 a^	130.0 (60.0, 394.0)	565.5 (332.0, 1771.0)	0.001
Wound Site ^b^			0.343
Lower extremity	9 (75.0)	14 (53.9)	
Upper extremity	3 (25.0)	8 (30.8)	
Back	0	4 (15.4)	
Exposed structures ^b^	5 (41.7)	11 (42.3)	0.970

^a^: median (25th, 75th percentile); ^b^: n (%); ^c^: missing value; ^d^: mean ± standard deviation; ^e^: will not add to zero due to some patients having multiple; CAD, coronary artery disease; HLD, hyperlipidemia; IFSG, intact fish skin graft; PVD, peripheral vascular disease; SM, synthetic matrix; TBSA, total body surface area burned.

**Table 2:** Simple linear regression for time to graftable wound bed.

	Parameter Estimate	Standard Error	*p* Value	VIF	r^2^
Initial Model					0.5624
Intercept	28.7290	3.4693	<0.0001	0	
IFSG	−15.6706	3.2614	<0.0001	1.3648	
Wound size	−0.0018	0.0010	0.0817	1.9319	
NPWT use	1.1316	3.9071	0.7385	2.0403	
Infection	13.3596	4.2133	0.0033	1.2046	
Final Model					0.5609
Intercept	29.6754	2.0072	<0.0001	0	
IFSG	−15.2508	2.9741	<0.0001	1.1654	
Wound size	−0.0021	0.0008	0.0172	1.2585	
Infection	13.5170	4.1323	0.0025	1.1898	

IFSG, intact fish skin graft; NPWT, negative pressure wound therapy; VIF, variable inflation factor.

## Data Availability

The data presented in this study are available on request from the corresponding author due to privacy restrictions.
